# Clinical characterization and possible pathophysiological causes of the Deventilation Syndrome in COPD

**DOI:** 10.1038/s41598-022-05118-w

**Published:** 2022-01-20

**Authors:** Mavi Dorothea Schellenberg, Sandra Imach, Gabriele Iberl, Marietta Kirchner, Felix Herth, Franziska Trudzinski

**Affiliations:** 1 Department of Pneumology and Critical Care Medicine, Translational Lung Research Center Heidelberg (TLRC-H), Member of the German Center for Lung Research (DZL), Thoraxklinik University of Heidelberg, Röntgenstraße 1, 69126 Heidelberg, Germany; 2 Institute of Biometry, Heidelberg University, Heidelberg, Germany

**Keywords:** Chronic obstructive pulmonary disease, Respiratory signs and symptoms

## Abstract

In daily routine, many COPD patients report early onset augmented dyspnea following use of NIV (Deventilation Syndrome, DVS) as a negative side-effect. The aim of this study is the clinical characterization and concrete definition of DVS. This monocenter prospective observational study collected demographic, physiologic and symptomatic data from 67 in-patients with severe COPD Gold III–IV and chronic hypercapnic failure before, during and after use of an established NIV. During their inpatient follow-up, we examined patients during the first hour after termination of nocturnal NIV. DVS was defined by the authors as an increase of ≥ 2 points on the Borg scale during the first 30 min in patients who reported repeated dyspnea after the use of NIV. We monitored cardiovascular and respiratory data and measured diaphragm excursion. Subjective dyspnea was documented by use of the Borg scale and questionnaires. In addition, respirator and demographic data were collected. DVS occurred in 58% of our COPD patient collective, showing predominant emphysema phenotype. Patients with DVS were more severely ill than non-DVS concerning bronchial obstruction (FEV1 0.6 vs. 0.8 l, *p* < 0.05) and hypercapnia during spontaneous breathing (pre NIV pCO_2_: 54.5 vs. 49.3 mmHg, *p* < 0.02). DVS patients showed significantly higher respiratory rates (RR) (20.1 vs. 18.1/min *p* < 0.05) after termination of NIV. This trial characterizes and defines early onset augmented dyspnea after the use of NIV, referred to as DVS. It is hereby brought to attention as a frequent side effect of long-term home ventilation and possible pathophysiologic mechanisms are elucidated.

## Introduction

Non-invasive positive pressure ventilation (NPPV, NIV) is a widespread therapeutic option for patients with chronic hypercapnic failure due to COPD^1,2^. Effectively reducing hypercapnia by high-intensity ventilation can not only lead to improvement of health-related quality of life and lung function status, but has shown to reduce hospital re-admission and prolong long-term survival^1^. Persistent improvements of dyspnea in the daytime have also been documented^3,4^.

Aside many positive aspects, there are also negative side effects of NIV. Most commonly described are dryness of the upper airways, abdominal bloating or sleep fragmentation^5,6^, but also multifaceted fear of therapy^7^.

This study now describes a further, highly burdening side effect: early onset acute dyspnea following the use of NIV—augmented in the morning, but also after use during the day.

During ongoing NIV, patients experience relief of dyspnea and can breathe without effort. In some patients, termination of NIV within minutes leads to increasing dyspnea and discomfort. This is accompanied by tachypnea with rapid, shallow breathing, increased heart rate, the need to sit upright (coachman's seat) and the feeling of restlessness, sometimes even leading to panic attacks and fear of use of NIV. This state is hereby defined as the Deventilation Syndrome (DVS).

The term "Deventilation Syndrome" in the context of dyspnea after mask removal was first described by Adler and colleagues^8^.

The authors hypothesized that in these patients, progressive hyperinflation, resulting from inappropriate ventilator settings, leads to patient-ventilator asynchrony (PVA) with a high rate of unrewarded inspiratory efforts and morning discomfort and identified inappropriate ventilator settings as a treatable cause of the problem in a group of 8 patients affected. Although this problem is common in clinical practice, there are no diagnostic criteria or recommended actions to address it. The aim of our study was to characterize DVS in patients with COPD and long-term home mechanical ventilation (LT-NIV) in order to better understand the pathophysiological background and by so to develop possible solutions for an improved care of the affected patients in the future.

## Methods

### Design

In a monocenter prospective observational study demographic, physiological and subjective data was collected from in-patients with severe COPD III–IV and chronic hypercapnic failure before, during and after use of an already established NIV.

Extended routine examinations included cardio-respiratory monitoring during and one hour after the nocturnal use of NIV, including ultrasonic diaphragm measurements. Subjective dyspnea was documented before and after use of NIV by use of the Borg scale and questionnaires were supplemented.

Being an observational study to document the Deventilation Syndrome, no study endpoints were defined. The authors refrained from power calculation due to the observational character of the study.

### Patients

Between November 2016 and January 2019, 71 patients with severe COPD (Gold III–IV) and established long-term home NIV who presented to our center for elective control of their ventilation at home were included in the study. Sufficient data was collected from 67 patients. Inclusion criteria were: men and women with stable COPD disease, GOLD stage III–IV, regular use (≥ 4 h/d) of NIV (prescription ≥ 1 year) and age > 18 years. Study participation required written consent and patients had to be able to follow the requirements of the project. The study protocol was granted permission by the ethics review board of the University of Heidelberg, Germany (number S-484/2016) and all patients gave their written informed consent. The DVS study was conducted in accordance with the Declaration of Helsinki. During the study any medically necessary concomitant therapy was allowed. Intercurrent diseases were treated according to the clinic standard. Exclusion criteria were: Acute pulmonary impairment (e.g. pneumonia), other acute diseases such as acute pulmonary artery embolism, hemoptysis, internal bleeding, pneumothorax. In addition, serious neurological disorders (e.g. stroke) or cardiovascular diseases with hemodynamic instability (e.g. heart failure NYHA ≥ III, myocardial infarction < 1 month ago) were excluded, as also newly occurring hypoxemia or worsening of hypercapnia ≥ 20% compared to previous values.

### Deventilation Syndrome (DVS)

DVS is determined by dyspnea—a highly subjective symptom. Being a novel syndrome, there is no definition of DVS to this date. Patients report recurring dyspnea episodes after the use of NIV, often preventing normal activities in the morning. This is accompanied by tachypnea with rapid, shallow breathing, the need to sit upright (coachman's seat) and the feeling of restlessness, sometimes even leading to panic attacks and fear of use of NIV. Objectification is best done by use of the modified Borg scale^9^. The minimal clinically importance difference (MCID) for Borg is 1 point^10^. For this study and further use of this terminology, DVS was defined by the authors as an increase of subjective dyspnea by ≥ 2 points on the Borg scale during the first 30 min after NIV termination—thus stressing the symptomatic and chronologic context after use of NIV. This threshold was determined in intention of clinical reflection of DVS, surpassing MCID of 1 point, but not excluding symptomatic patients who require symptom management.

### Ethics approval

Ethics approval by Ethics Committee University of Heidelberg, S-484/2016.

## Measurements

### Survey examination timeline

**Figure 1:**
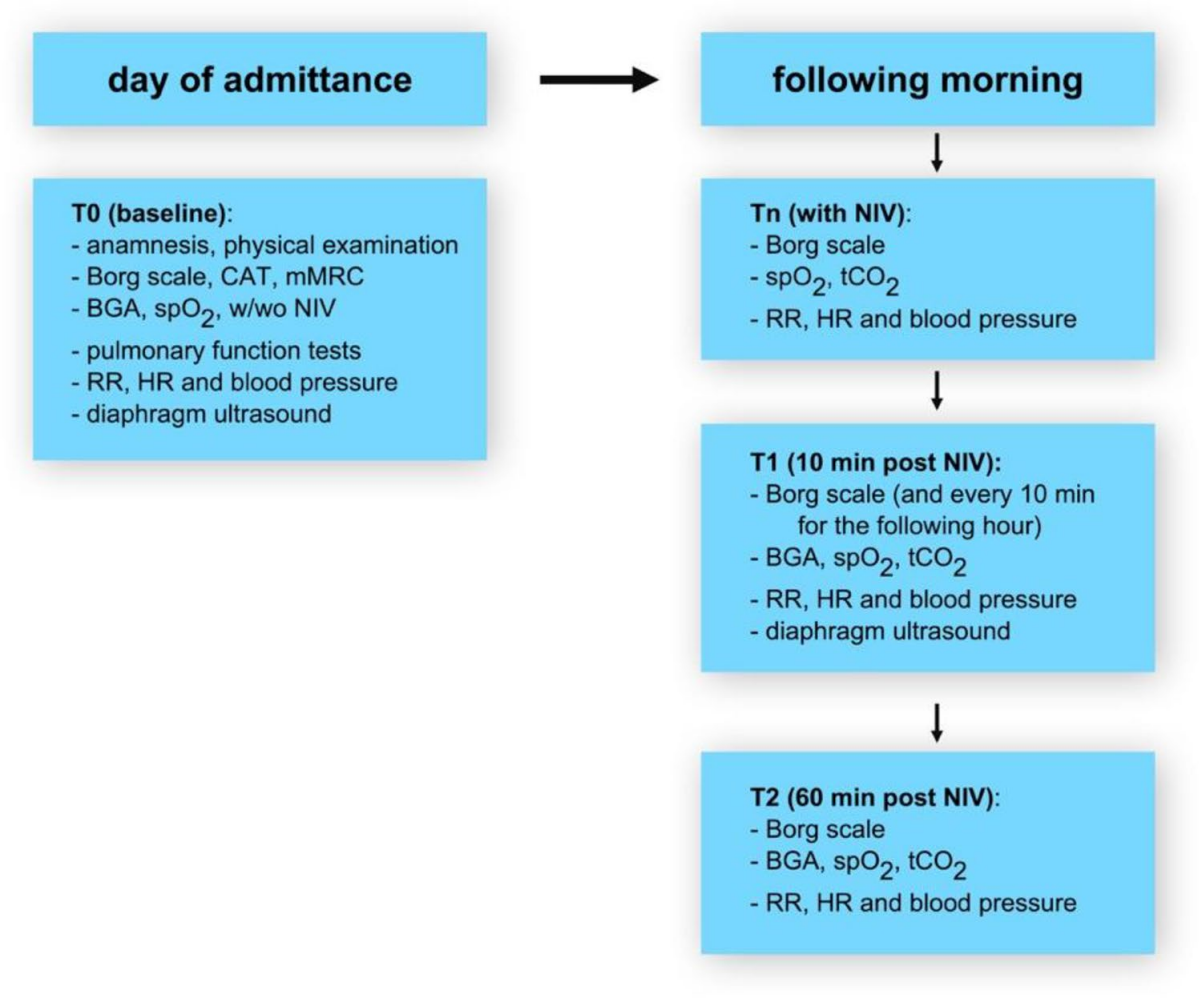
Timeline of the study.

During the study, medical history, questionnaires, vital signs, and functional examinations, including diaphragmatic ultrasound, were collected at various time points. The timeline of the study is shown in Fig. 1.

### Pulmonary function diagnostics

These were carried out according to the internal standards of the lung function department of the University Thoraxklinik Heidelberg, using MasterScreenBody and PFT Body + Diff. by Care Fusion (Jaeger).

Body plethysmography: Documentation of body plethysmography (functional residual capacity (FRCpleth), specific airway resistance (sRaw), total lung capacity (TLC) and residual volume (RV), airway resistance was calculated by use of sRaw and FRC(pleth)) and spirometric data (vital capacity (VC), forced vital capacity (FVC), forced expiratory volume (FEV1) and percentage value FEV1/FVC index . Furthermore, measurement of diffusion capacity (TLCO) was documented.

Reference values were calculated according to GLI for spirometric parameters and TLCO^11,12^, while ECSC was referenced for body plethysmography^13^.

Respiratory muscle strength: capacity of respiratory muscles was measured non-invasively by maximal inspiratory mouth pressure (Pimax). Respiratory drive was measured indirectly by occlusion pressures (P0.1)^14^.

### Ultrasonic diaphragm measurement

Measurements of diaphragm excursion were performed using the Philips Ultrasound Sparq with an abdominal transducer C 6-2. Patients were examined during spontaneous breathing on admission day (T0) and the following morning after termination of NIV (T1). This was done in a dorsal lying position, the torso elevated at approx. 45°. The right diaphragm was located in B-Mode and then excursion measured in M-Mode subcostal, mid clavicular line^15,16^. Pre and post NIV measurements on one patient were performed by the same examiner, two people in whole performed the exams by standardized procedure.

### Measurement of carbon dioxide values and blood gas analysis

Carbon dioxide values were collected by two methods: continuous transcutaneous measurement and capillary blood draw (blood gas analysis, BGA), both examined on the earlobe.

BGA was assessed by arterialized capillary ear-lobe puncture earlobe skin^17^. The ABL 800 flex from Radiometer GmbH was used as blood gas analyzer. Transcutaneous CO_2_ measurement was performed using TOSCA transcutaneous monitor TCM 4, Radiometer during the night and 60 min after NIV termination. NIV parameter adjustments were performed when needed according to increased hypercapnia levels.

### Monitoring

Nightly monitoring of patients undergoing long term home NIV is an essential part of NIV surveillance, reflecting the respiratory situation and efficiency of ventilation during and after sleep (RR, HR, BP, spO_2_, CO_2_). This standardized procedure was performed during nocturnal use and extended one hour after termination of NIV on our ward using Philips IntelliVue MX550 and TCM monitors. (Results spO_2_/HR/BP see supplementary information).

### Questionnaires

#### Borg perception of exertion CR10 scale

Acquired by modified Borg scale (MBS) with 10-point dyspnea assessment^9^. An increase of ≥ 2 points after NIV termination was defined by the authors as relevant for DVS.

CAT: COPD Assessment Test

Cut off: < 10 = low impairment to > 30 = very high impairment^18^.

mMRC: Modified Medical Research Council

Score from 0 ("never breathless, except during intense exertion") to 4 ("too short of breath to leave the house or to dress and undress alone")^19^.

### Statistical analysis

Baseline characteristics are presented for the whole sample and stratified by group (DVS vs. nDVS) by mean and standard deviation (SD) or median and interquartile range (IQR) or frequencies (n, %) as appropriate. Differences between groups were investigated by means of independent t-test, Mann–Whitney U test or chi-square test retrospectively. Continuous variables were explored graphically to justify parametric analysis.

Longitudinal models were applied to test for time × group effects for outcomes measured over time. Except for BORG (ordinal scale), a linear regression model with repeated measures statement was applied for each outcome with the explaining variables time, group, and interaction term. Group and time effects with confidence intervals and *p* value were presented based on the contrast between groups at each visit and contrasts between the visits for each group.

For Borg, the total score was analyzed descriptively by median (IQR) at the four measurement points in time for each group (DVS and nDVS).

To analyze the relationship of Borg and FEV1, Spearman correlation coefficients were calculated.

This is an exploratory analysis so that *p* values are interpreted descriptively. A *p* value < 0.05 was considered statistically significant. All analyses were performed in SAS version 9.4.

## Results

### Baseline characteristics

Between November 2016 and January 2019, 71 patients were included in the study. One patient was excluded due to double enrollment, three patients did not complete the Borg scale, therefore 67 patients were further evaluated. 39 patients were retrospectively diagnosed with DVS, 28 did not present DVS symptoms.

Enrollment included overall 28 males and 39 females (corresponding to 42% and 58% respectively). The mean (SD) age of all patients was 66.1 ± 6.7 years. Comorbidities were widespread, but severe and/or acute diseases were excluded according to the exclusion criteria of the study protocol. It is noteworthy that our COPD collective requiring NIV showed a predominant emphysema phenotype.

No serious complications occurred during the study, e.g. acute exacerbation of the disease. All patients were compliant with both participation in the study and with their NIV therapy. However, 5 patients did not wish to perform lung function tests. In all, 13 patients were unable to undergo or complete body plethysmography due to their physical constitution and extremely high lung inflation, one further patient had non legible RV rates. Interestingly, 11 of these 13 patients were retrospectively diagnosed with DVS.

### Ventilatory setting

Ventilatory settings (VS) were documented (Table 1). The majority of patients was ventilated in ST mode (spontaneous/timed mode), 2 patients of the cohort used an aPCV mode (assisted pressure-controlled ventilation).

**Table 1:** Subgroup baseline characteristics.

	DVS, n = 39 Mean ± SD, n (%)	nDVS, n = 28 Mean ± SD, n (%)	*p* value*
Weight kg	67.3 ± 21.8	71 ± 20	0.485
Height cm	166.6 ± 9.8	165.6 ± 8.5	0.654
Age years	66.4 ± 6.6	65.9 ± 7	0.765
Male	18 (46.2%)	10 (35.7%)	0.39
Female	21 (53.8%)	18 (64.3%)	
Pack-years	52.9 ± 26.3	39.8 ± 20.2	0.031
**COPD pharmacotherapy**			
SAMA	23 (59%)	15 (54%)	0.660
LAMA	39 (100%)	28 (100%)	–
SABA	35 (90%)	21 (75%)	0.108
LABA	36 (92%)	25 (89%)	0.669
ICS	24 (62%)	13 (46%)	0.220
Opiods	11 (28%)	3 (11%)	0.082
BMI kg/m^2^	24.1 ± 7.2	25.8 ± 6.7	0.323
Vital capacity liters	1.8 ± 0.70	2.0 ± 0.74	0.195
Total lung capacity liters	8.1 ± 1.9	7.4 ± 1.7	0.102
Total lung capacity %pred	144.9 ± 35.4	134.3 ± 20.8	0.668
FEV1 liters	0.63 ± 0.3	0.81 ± 0.3	0.016
FEV1%	25.2 ± 10.1	33.2 ± 13.5	0.009
FEV1/FVC index %	36.9 ± 9.5	40.8 ± 12.3	0.167
Pimax kPa	4.0 ± 1.8	5.0 ± 2.1	0.064
P0.1 kPa	0.7 ± 0.3	0.6 ± 0.2	0.258
RV liters	6.1 ± 1.6	5.4 ± 1.8	0.100
RV %	281.2 ± 78.2	246.1 ± 71.9	0.097
CAT points	25.5 ± 6.8	19.8 ± 7.5	0.002
MMRC	4.0 ± 1.2	3.0 ± 1.4	0.02
DM pre NIV (T0) mm	1258 ± 545	1284 ± 588	0.837
DM post NIV (T1) mm	1102 ± 485	1025 ± 417	0.549
Difference T0–T1 mm	156 ± 500	259 ± 491	0.41
**Ventilatory settings**			
IPAP, max, cm H_2_0	21.5 ± 3.2	20.8 ± 3.3	0.969
PEEP, cm H_2_0	5.2 ± 0.9	5.2 ± 0.8	0.410
RR, per minute	11.1 ± 2.0	10.7 ± 2.3	0.464
Ti_min, seconds	0.9 ± 0.1	0.9 ± 0.1	0.336
Ti_max, seconds	1.6 ± 0.2	1.6 ± 0.2	0.893
O_2_, l/min	1.9 ± 0.7	1.4 ± 1.0	0.007

*p value based on t-test for continuous variables and on chi-square test for categorial variables.

### Subgroup characteristics—DVS versus nDVS

Subgroup characteristics are presented in Table 1.

### Lung function analysis

Subgroup analysis of lung function measurements showed higher bronchial obstruction in patients with DVS and a trend to higher lung inflation. Lung inflation and respiratory muscle drive (P0.1) were elevated and respiratory muscle capacity (Pimax) was reduced in the DVS group compared to nDVS, though not statistically significant (*p* > 0.05) due to high drop-out rate in body plethysmography (insufficient maneuver). See Table 1, Supplement Fig. 6–7 (SF 6–7).

### Carbon dioxide levels

When analyzing subgroup data, focus was placed on transcutaneous CO_2_ measurements. As there was no transcutaneous T0 data, capillary CO_2_ measurements were alternatively respected.

Patients with DVS had higher CO_2_ levels than nDVS patients at all times of measurement (Table 2). Hypercapnia was decreased during NIV in both subgroups, but remained higher in DVS. This effect was even clearer after NIV termination at T1 and T2.

**Table 2:** Subgroup tCO_2_ levels at different points of time.

	CO_2_ mean ± SD DVS	CO_2_ mean ± SD nDVS	*p* value
T0 (p) mmHg	54.5 ± 11.1	49.4 ± 9.5	0.023
Tn (t) mmHg	49.1 ± 7.5	45.1 ± 7.3	0.050
T1 (t) mmHg	53.9 ± 9.2	47.8 ± 8.4	< 0.01
T2 (t) mmHg	53.4 ± 8.8	47.3 ± 7.4	< 0.01

T0 baseline: no NIV Tn: with NIV (≥ 4 h use) T1: 10 min after NIV T2: 60 min after NIV.

p: partial pressure, pCO_2_; t: transcutaneous, tCO_2_.

### Respiratory rate, subgroups

**Figure 2:**
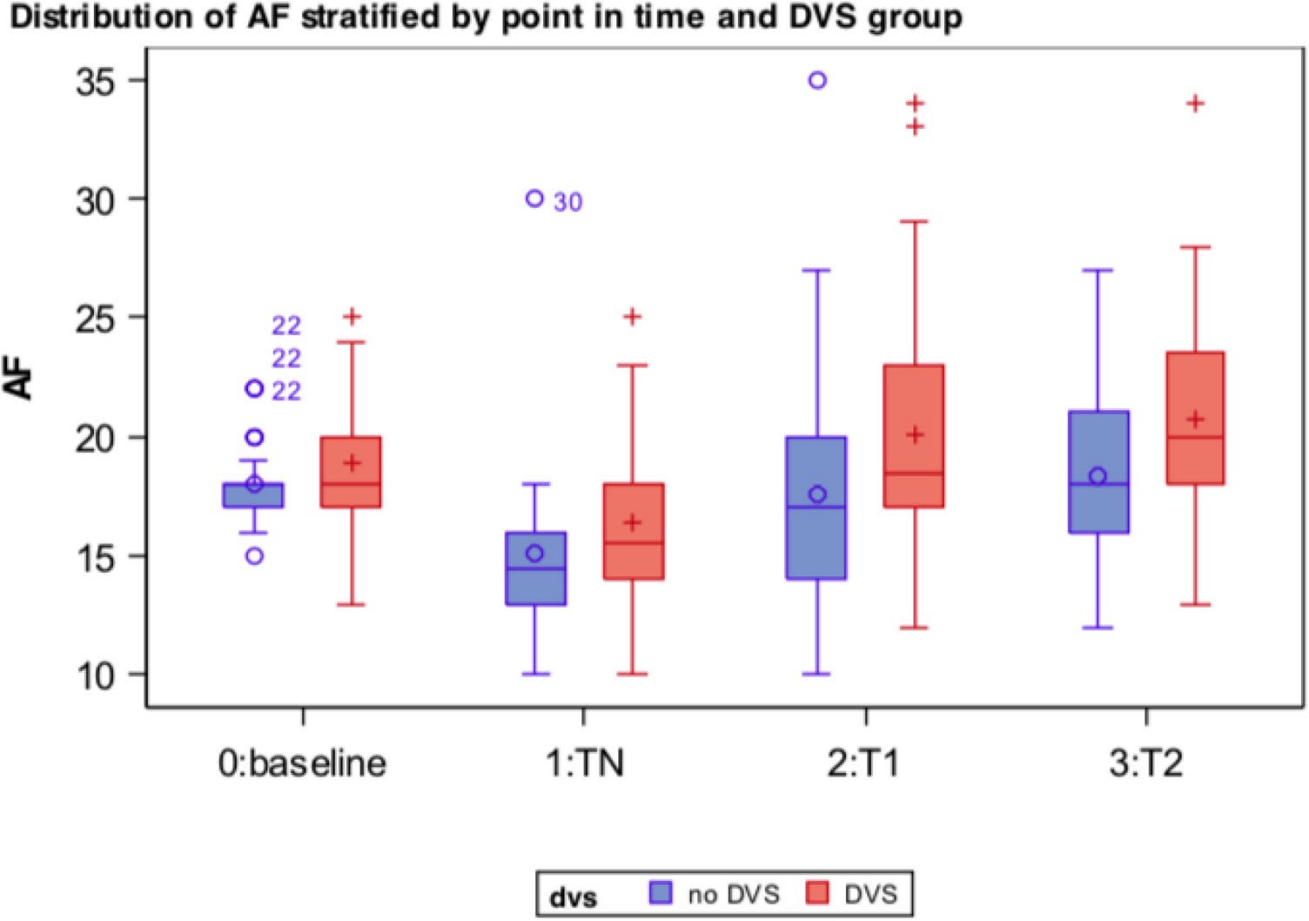
Respiratory rate in subgroups DVS/nDVS stratified by time.

Respiratory rate (RR, breaths per minute (bpm)) was measured by continuous monitoring at four points of time (Fig. 2). It decreased significantly in the DVS group during use of NIV compared to spontaneous breathing. RR increase after termination of NIV was very clear and persisted up to one hour later. There was no significant difference in means between the RR at admission to after NIV termination.

Analysis in the nDVS group also showed a similar pattern with deceleration during and acceleration after use of NIV.

Subgroup comparison (Fig. 2,) of RR on the day of admission and undergoing ventilation showed no significant differences in means (*p* > 0.1), though a trend towards tachypnea in the DVS group was documented. Subgroup comparison after termination of NIV (T1) showed significant differences: mean RR in the DVS group was higher than nDVS. This effect did not persist at T2, though further marked a trend. See Fig. 2 (and Supplement: Table ST1).

### Oxygen

There were no significant differences in oxygen saturation, both as independent variables at the different points in time and in a group as a dependent variable (Supplement: Table ST2, Fig. SF1).

### Ultrasonic measurement of diaphragm excursion

The ultrasonic examination of the diaphragm was performed in all patients on day of admittance (T0) and the next morning after NIV termination (T1). In the overall group, T0 mean diaphragm excursion (± SD) was 1269 ± 559 mm, at T1 1069 ± 456 mm. Excursion difference between T0 and T1 (mean −200 mm) was statistically significant (*p* 0.03).

Inter-subgroup analysis showed a smaller difference of excursion rates between T0 and T1 in the DVS group compared to nDVS, though not statistically significant.

### Borg scale CR10

67 patients correctly filled out the Borg scale.

For this trial, an increase of ≥ 2 points on the Borg scale within 30 min after NIV termination was defined as Deventilation Syndrome, based on the patient’s subjective dyspnea.

**Figure 3:**
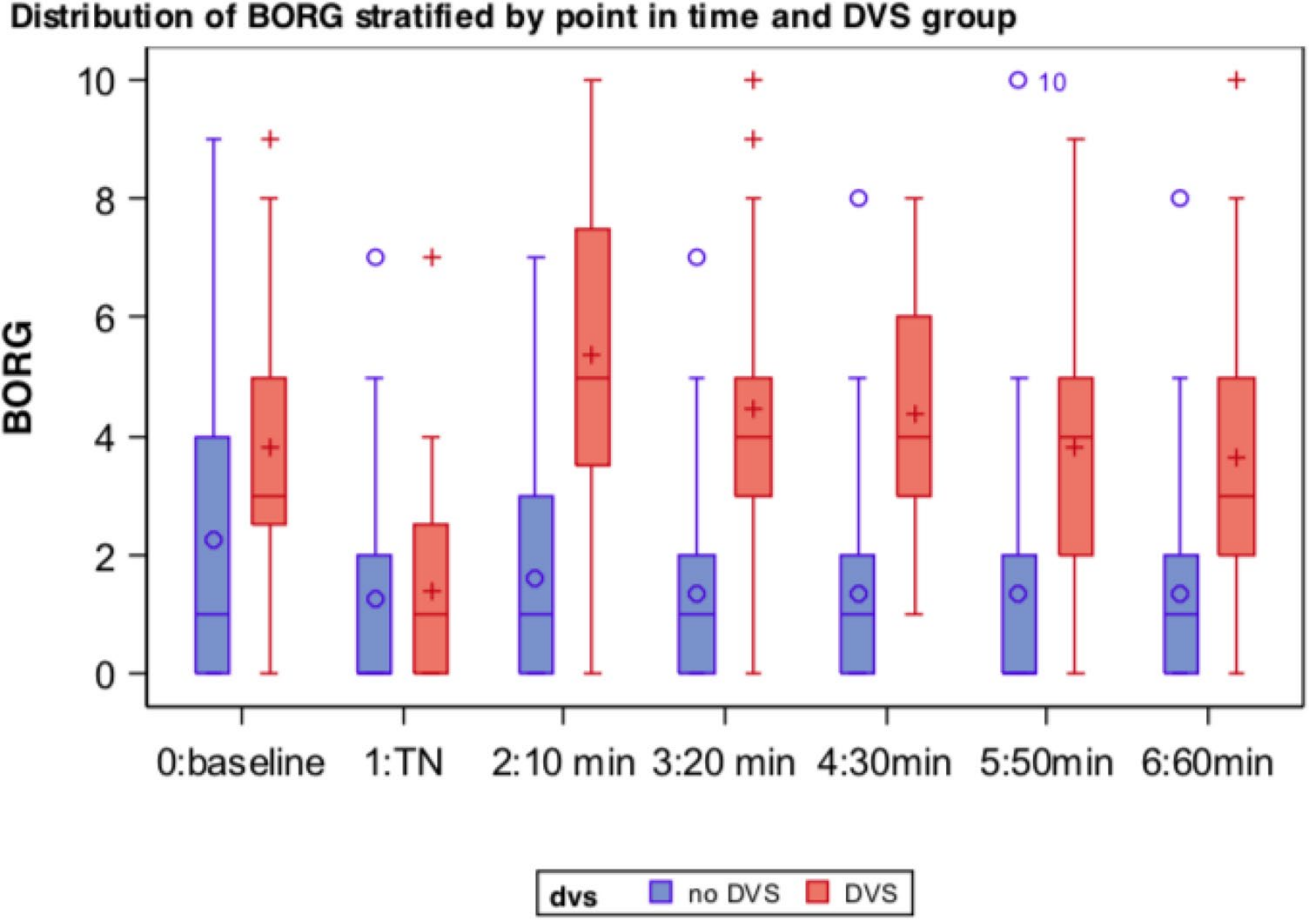
Borg stratified by point of time and subgroup.

This criterion was met by 39 out of 67 patients, corresponding to a share of 58.2%. The majority of patients experienced dyspnea immediately after NIV termination (≤ 10 min), only 3 patients showed symptoms between 10 and 30 min. In the DVS group, baseline (T0) median (IQR) Borg score was 3.0 (3.0) points and dropped to 1.0 (3.0) points undergoing NIV (Tn). After NIV termination, Borg value increased to median of 5.0 (4.0) points during the first 10 min, over 90% of the DVS patients experienced greater respiratory distress in this first time segment. Within the next hour, median Borg values dropped continuously and settled after 60 min at 3.0 (3.0) points—thus meeting baseline levels (Fig. 3).

Patients without DVS showed very low variance of dyspnea and overall lower Borg scores (Fig. 3).

### Borg scale and FEV1

There was a high correlation of Borg scale rankings and the severity of pulmonary impairment, measured by FEV1. Lower FEV1 levels were associated with more dyspnea (*p* < 0.01).

### CAT

The CAT questionnaire documented a higher mean score in DVS patients than nDVS, reflecting lower FEV1 levels and higher disease burden. This effect was significant *p* < 0.01 (Table 1).

### MMRC

The mMRC questionnaire revealed a significantly higher score in DVS than nDVS (median (IQR) 4.0 (2.0) vs. 3.0 (3.0) points, maximum of 4 points (0–4), *p* 0.02), reflecting higher respiratory distress in everyday life (Table 1).

## Discussion

Acute dyspnea after the use of non-invasive ventilation (NIV) in a long-term setting is a common and stressful symptom in COPD patients. This is the first study to clinically characterize the Deventilation Syndrome (DVS) and suggest routine documentation of patient dyspnea and discomfort after the use of NIV as well as the objectified increase of dyspnea by > 2 points (Borg scale). Despite an abundance of publications on NIV in COPD, many also addressing negative side-effects, DVS has remained widely unaddressed.

DVS was identified in 58% of COPD patients with chronic hypercapnic failure in a routine in-patient setting, thus emphasizing the relevance of the subject. Ventilator settings were effective in both groups (DVS and nDVS), leading to significant decrease of CO_2_ levels and documented reduction of dyspnea per Borg scale undergoing NIV. Both groups were fully adapted to the regular use of NIV and showed no signs of discomfort or asynchronicity undergoing therapy. Nightly cardiovascular and pulmonary monitoring showed no signs of relevant sleep disordered breathing.

Dyspnea is a complex symptom and still not fully understood, though its role as a predictive marker in chronic lung diseases has been acknowledged^20–22^. It is highly subjective in perception and does not necessarily correlate with specific tests (e.g. spirometry, blood gas analysis), but dyspnea can—and should—be measured. Underlying mechanisms and qualities of dyspnea can be categorized in:

elevated work or effort in breathing (elevated mechanical load), tightness of the chest (bronchoconstriction) and intensity of air hunger (increased inspiratory drive, desensitized pulmonary mechanoreceptors)^22^. Following these main mechanisms can help understanding causes and thus provide therapeutic concepts in DVS. Ultrasonic measurement of the diaphragm showed significant (pre NIV 1269 mm, post-NIV 1069 mm, *p* 0.03) movement impairment after the use of NIV in the overall group, possibly expressing transient reduction of diaphragm muscle strength due to hyperinflation after use of NIV and diaphragm displacement. Bed-side lung function tests were not performed, but will be added in further trials. Variability of diaphragm movement was overall very high, though pre and post NIV examinations were performed by the same observer. There was no significance between each subgroup, though the differences of diaphragm movement pre and post NIV do strike as noteworthy (nDVS 260 mm vs. DVS 157 mm). Measurements of respiratory muscle strength via Pimax (from RV) were reduced in the DVS group compared to nDVS (4.0 vs. 5.0 kPa, *p* 0.06). Further assessment of the diaphragm function by ENMG or SNIP was not conducted in this trial but should be considered in further studies.

While diaphragm impairment seems to be well tolerated in some patients, it could pose the fundament of DVS—especially in context of accelerated respiratory rates. Changes of the respiratory rate (RR) provide a further, if not the main key in understanding DVS. Patients in both subgroups showed similar patterns of the RR: a significant drop from baseline to undergoing NIV—then picking up again back to baseline level (and exceeding baseline in DVS) after the use of NIV. Remarkably, dyspnea by Borg scale and RR did not differ in both groups at baseline (afternoon) measurement on admission day or undergoing NIV, thus showing a similar initial patient situation.

DVS shows an impact after use of NIV: in DVS, RR was higher than nDVS patients (T_10min_: 20.1 vs. 18.1 bpm, *p* 0.04) and remained elevated during the hour of monitoring (T_60min_: 20.4 vs. 18.9 bpm, *p* 0.1, though most DVS patients were subjectively recovered by Borg scale 60 min after NIV termination in this study. RR after use of NIV in DVS patients was higher than at T0 (baseline) although not statistically significant, so time adjusted RR change should be acknowledged. During the short period of 10 min post NIV, DVS patients showed a mean RR increase of 4.3 bpm, in nDVS 2.8 bpm.

Potential reasons for acute RR increase could be reduced diaphragm strength or elevated inspiratory drive due to higher CO_2_ levels in the DVS group (tCO_2_ T_10min postNIV_ 54 vs. 48 mmHg, *p* < 0.01). Also, higher overall bronchial obstruction in DVS (FEV1 25% vs. 32.5% nDVS, *p* 0.01), furthermore augmented obstruction in the early morning hours (circadian, vagal stimulation, decreased medication levels), increases breathing muscle activity and effort and can trigger dynamic hyperinflation in already severely inflated lungs (RV DVS 6.1 vs. nDVS 5.4 L, *p* 0.1), thus circling into rapid shallow breathing (RSB). RSB and impaired diaphragm function have been identified as predictors for weaning failure^23^, reflecting relevant breathing muscle exhaustion. A possible negative influence of DVS on long-term prognosis of COPD patients with NIV is yet to be surveyed. It is also noteworthy that many DVS patients expressed fear of NIV termination in the morning due to experienced dyspnea in the past, posing a further possible cause for RR acceleration.

Furthermore, nocturnal patient-ventilator asynchrony (PVA) can currently not be ruled out as a conceivable trigger, as polygraphy was not carried out.

Higher levels of hypercapnia in patients with DVS are probably an expression of higher disease burden, as seen in the lung function tests—though underventilation with NIV cannot be fully ruled out.

Oxygen levels at any time also did not differ in both groups and thereby remain disregarded.

There are limitations in this trial to be discussed. First, assessment of diaphragm function was performed only by ultrasonic diaphragm excursion (DE) and only in two examinations, showing high standard deviation. Initially, DE was only thought an addendum to other focus topics—it now evolved to possibly one of the key features in DVS. In the future, more detailed data (e.g. diaphragm thickening index, repeated measurements) will be needed. Second, there was no guideline in the protocol concerning medication intake. This was intentionally put in the hands of each patient to decide by need, hence heterogenous treatment patterns. Subsequent trials should standardize this procedure. Third, monitoring was only continued for one hour during which some patients did not return to baseline level in terms of dyspnea, respiratory rate and CO_2_ levels. So, full duration of DVS has not yet been captured. Fourth, polygraphy was not initiated, which would undoubtedly enhance the data and illuminate possible patient-ventilator asynchrony. And fifth, true prevalence was not documented, as not all in-patients were screened for this study.

Despite many limitations in this trial, important data has been collected and helped establish potential key points concerning DVS. Therapy concepts have been established in our clinic and will be the focus of further trials.

## Conclusion

In conclusion, DVS is a frequent, but to this day insufficiently regarded side effect of LTH-NIV. The recognition of DVS is of high clinical relevance for health care givers and patients. This trial offers not only definition of DVS, but also documents frequency of occurrence and relevant patient data leading to concepts of factors that presuppose DVS, by this stepping towards prevention and therapy of this often tormenting symptom. Further research is required to substantiate DVS and establish alleviating therapies.

## Supplementary Information

Supplementary Information.

## Data Availability

The datasets used and/or analyzed during the current study are available from the corresponding author on reasonable request.

## Abbreviations

BGA: Blood gas analysis
BP: Blood pressure
Bpm: Breaths per minute
CHRF: Chronic hypercapnic respiratory failure
COPD: Chronic obstructive pulmonary disease
DE: Diaphragm excursion
DVS: Deventilation Syndrome
ENMG: Electroneuromyography
EPAP: Expiratory positive airway pressure inspiratory/expiratory ratio
HR: Heart rate
IPAP: Inspiratory positive airway pressure
kHz: Kilohertz
kPa: Kilopascals
LTH-NIV: Long term home non-invasive ventilation
Mbar: Millibar
MmHg: Millimeter of mercury
nDVS: Non Deventilation Syndrome
NIV: Non-invasive ventilation
NPPV: Non-invasive positive pressure ventilation
pCO_2_: Arterial partial pressure of carbon dioxide
PE: Physical exam
PEEP: Positive end-expiratory pressure
tCO_2_: Transcutaneous measurement of arterial partial pressure of carbon dioxide
PVA: Patient-ventilator asynchrony
RR: Respiratory rate
RSB: Rapid shallow breathing
SNIP: Sniff nasal inspiratory pressure
SpO_2_: Oxygen saturation of hemoglobin measured by pulse oximetry
TI_MAX_: Maximal inspiratory time
VE: Minute ventilation
V_T_: Tidal volume
