# Supplementary material for: Clinical characterization and possible pathophysiological causes of the Deventilation Syndrome in COPD
Source: Sci Rep. 2022 Jan 20;12:1099. doi: 10.1038/s41598-022-05118-w (PMC8776740; doi:10.1038/s41598-022-05118-w)

**Supplementary Information**

**Clinical Characterization and Possible Pathophysiological Causes of the Deventilation Syndrome in COPD**

Mavi Schellenberg^1^, Sandra Imach^1^, Gabriele Iberl^1^, Marietta Kirchner^2^, Felix Herth^1^, Franziska Trudzinski^1^

^1^Department of Pneumology and Critical Care Medicine, Thoraxklinik University of Heidelberg, Translational Lung Research Center Heidelberg (TLRC-H), Member of the German Center for Lung Research (DZL), Heidelberg, Germany

^2^Institute of Medical Biometry Heidelberg University, Germany

Corresponding author:

Dr. med. Mavi Dorothea Schellenberg

Thoraxklinik University of Heidelberg

Röntgenstraße 1, 69126 Heidelberg, Germany, Email: mavi.schellenberg@med.uni-heidelberg.de

**Respiratory rate**

*ST1: Subgroup analysis respiratory rate (RR) at different points in time*

|  | **RR DVS** | **RR nDVS** | **P value** |
| --- | --- | --- | --- |
| T0 n/minute | 18.9±2.6 | 18.0±1.9 | 0.398 |
| Tn n/minute | 16.4±3.7 | 15.3±3.5 | 0.260 |
| T1 n/minute | 20.1±5.3 | 18.1±5.2 | 0.046 |
| T2 n/minute | 20.4±3.6 | 18.9±4.8 | 0.128 |

*T0 baseline: no NIV Tn: with NIV (≥4h use) T1: 10 minutes after NIV T2: 60*

**Oxygen**

*ST2: Subgroup analysis spO_2_ at different points in time*

|  | DVS, n=39  mean±SD, spO_2_ (%) | nDVS, n=28  mean±SD, spO_2_ (%) | p-value* |
| --- | --- | --- | --- |
| T0 (baseline) | 95.0±2.9 | 95.7±2.3 | 0.454 |
| Tn (NIV) | 96.3±2.5 | 96.4±3.0 | 0.895 |
| T1 (10min post NIV) | 94.3±4.7 | 95.8±2.2 | 0.107 |
| T2 (30min post NIV) | 95.4±3.0 | 94.9±6 | 0.581 |

*p-value based on t-test for continuous variables and on chi-square test for categorial variables

*SF1: Subgroup analysis spO_2_ at different points in time*


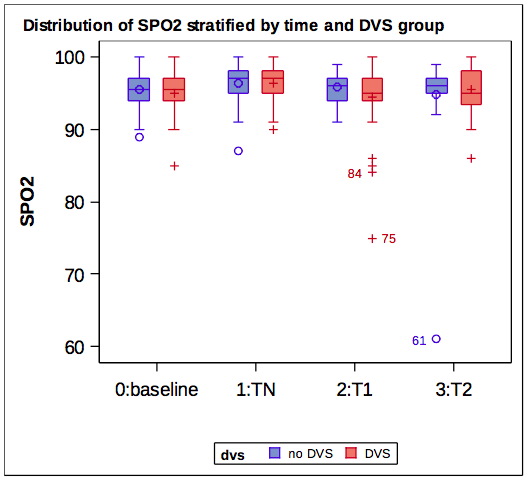


*T0 baseline: no NIV Tn: with NIV (≥4h use) T1: 10 minutes after NIV T2: 60*

**Circulatory System**

*Cardiovascular changes during and after use of NIV were documented, both heart rate and blood pressure dropped during (Tn) and elevated after NIV (T1 and T2). Measurements were noted at Tn in wake patients; therefore, this was not a sleep induced phenomenon. Interaction of positive pressure ventilation and the cardiovascular system have often been recorded, especially in the use of PEEP. In this trial, we did not focus on possible cardiovascular causes of augmented dyspnea, though this surely should be addressed in further studies.*

Heart rate

We documented a significant decrease in heart rate (HR) in both subgroups during the application of NIV (DVS: T0: HF 85.3/min vs Tn: HF 79.6/min, p<0.01; nDVS: T0: HF 78.3/min vs Tn: HF 72.1/min, p 0.01).

After NIV termination in the DVS group, HR increased and surpassed baseline measurements (T1: 86/min, T2: 89/min, p<0.01). In the nDVS group, there was a slight increase after NIV termination, but without significant change to the baseline rates (T0 78.3 vs T1 76.2 and T2 81.5/min).

Interestingly, DVS patients showed a significantly higher HR at all times compared to nDVS (p<0.05).

*SF2: Overall analysis of heart rate (HF) at different points in time*


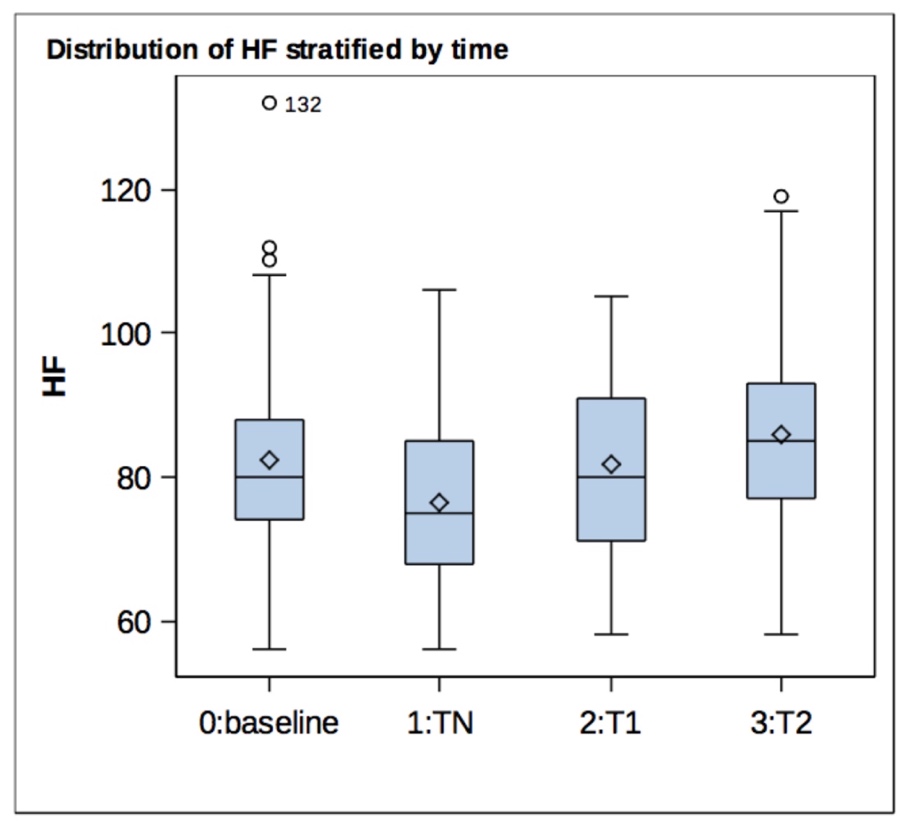


*SF3: Subgroup analysis of heart rate (HF) at different points in time*


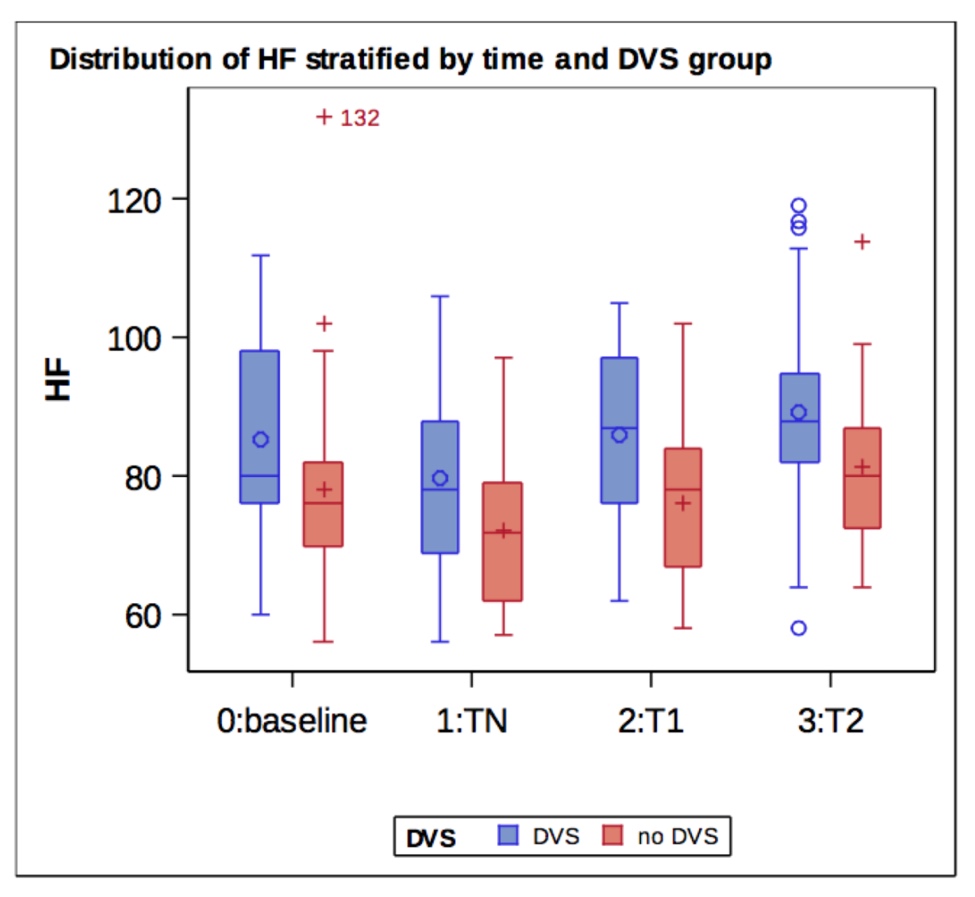


Blood pressure

In the DVS group, systolic blood pressure (BP) rates showed a significant decrease from baseline to undergoing NIV (T0 125 vs Tn 117mmHg p 0.04) and an increase after NIV use (Tn 117 vs T1 127 and T2 130mmHg p 0.01 and <0.01). The increase of systolic BP returned to baseline niveau and did not surpass.

The nDVS group showed no effect regarding systolic BP rates.

Diastolic BP rates showed no statistical effect in either group.

*SF4: Overall analysis of systolic blood pressure (RR) at different points in time*

**
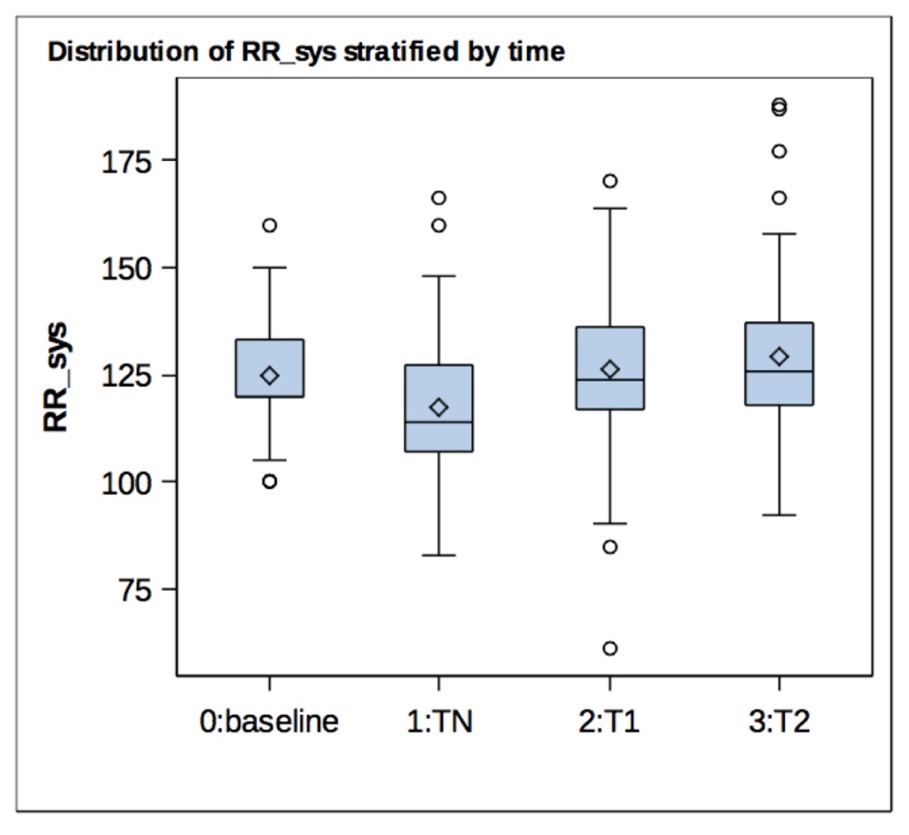
**

*SF5: Subgroup analysis of systolic blood pressure (RR) at different points in time*

**
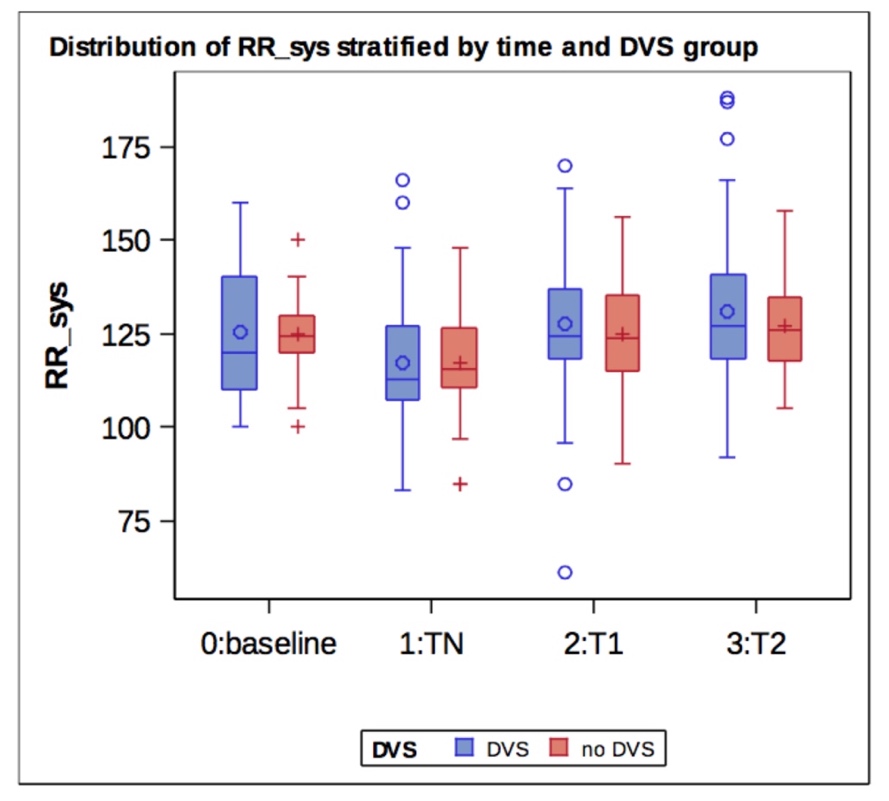
**

**Lung function analysis**

*SF6: Residual volume (RV, liters) stratified by DVS group*


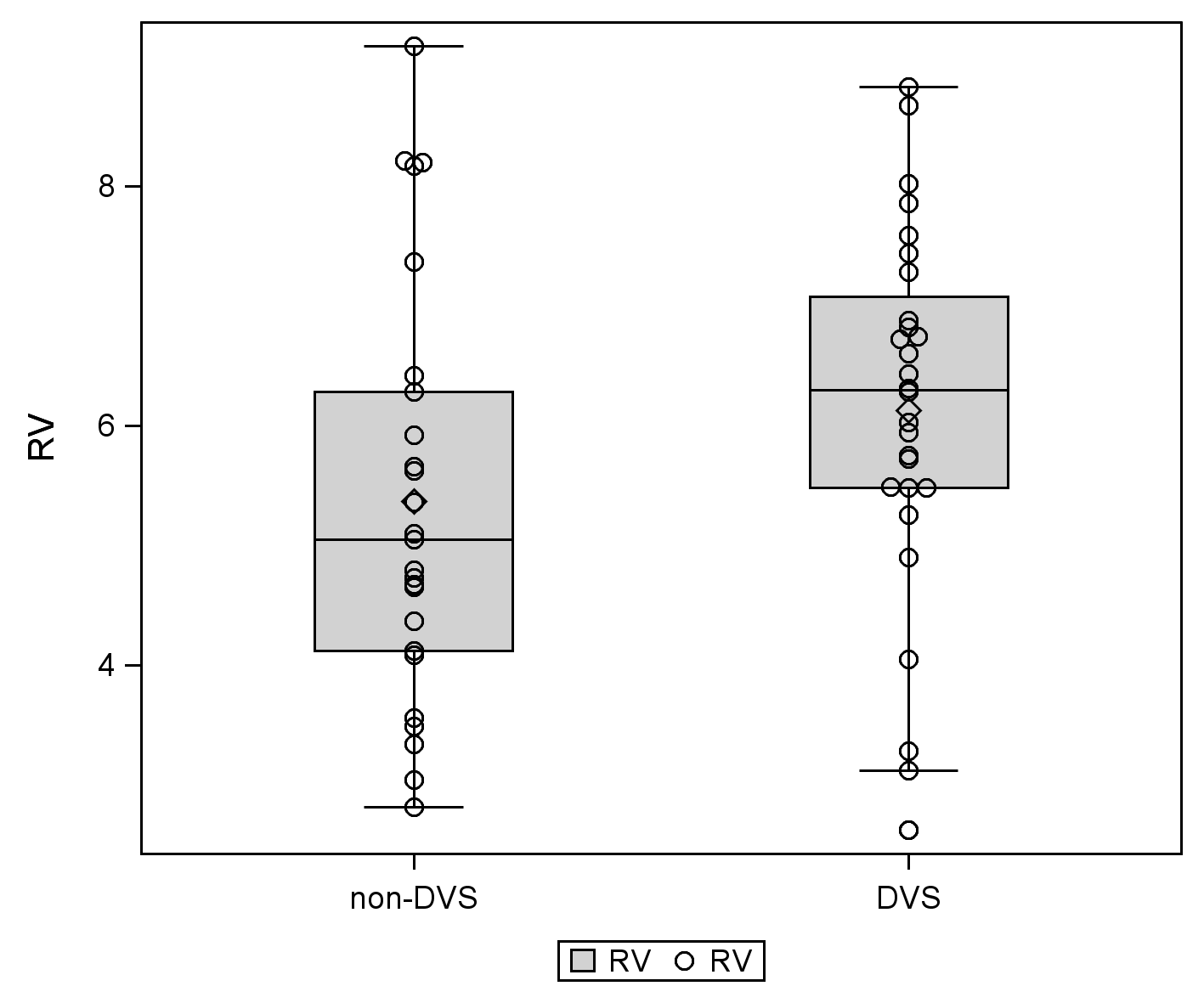


*SF7: Residual volume (% predicted) stratified by DVS group*


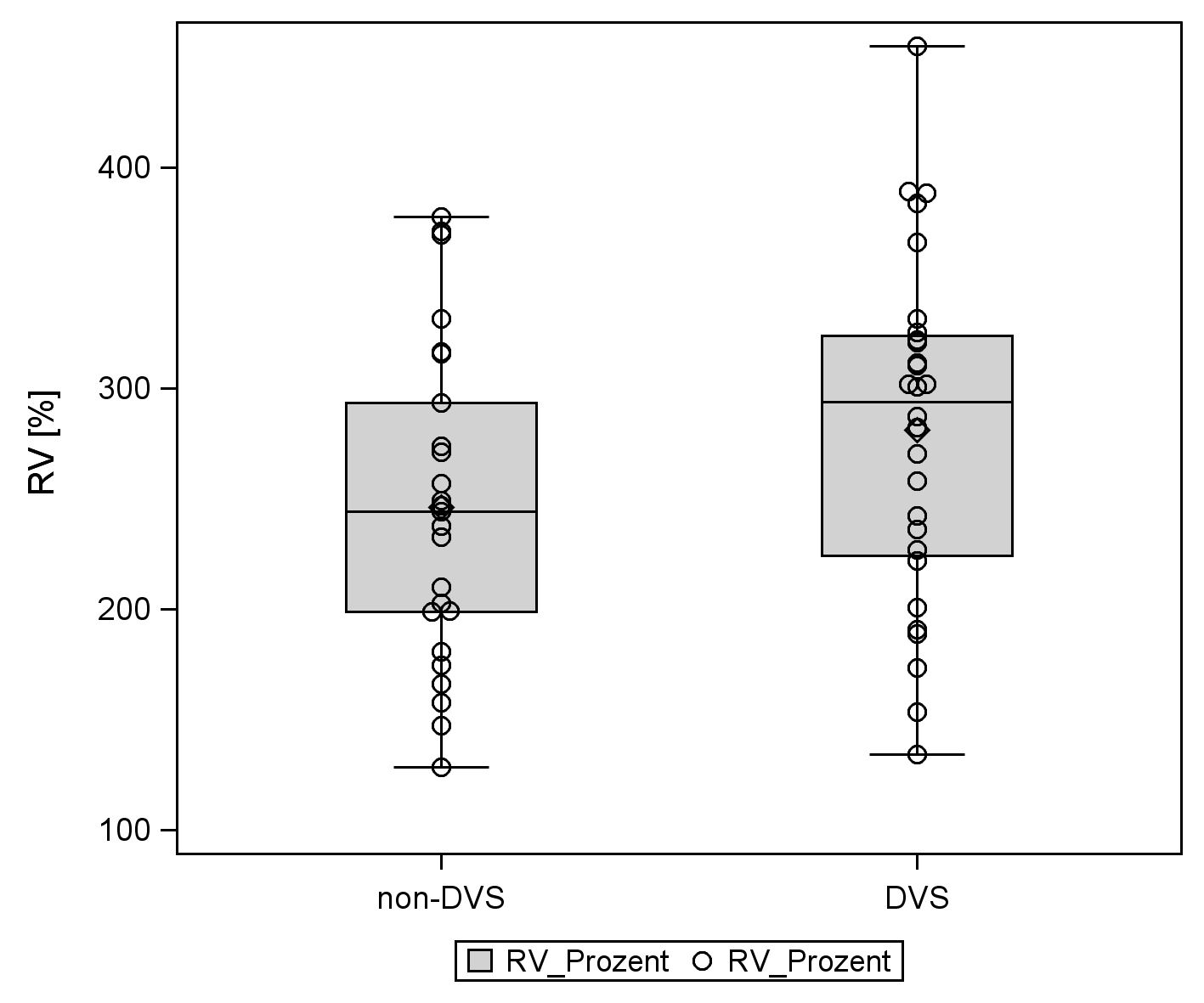

Supplement: Supplementary file 1 — Supplementary Information. [file 41598_2022_5118_MOESM1_ESM.docx]
